# Eliminating tuberculosis by addressing access and equity

**DOI:** 10.1016/j.lansea.2024.100520

**Published:** 2024-12-10

**Authors:** The Lancet Regional Health – Southeast Asia

WHO released its Global Tuberculosis Report recently, formally recording the return of tuberculosis (TB) as the world's leading infectious disease killer in 2023, 3 years after being replaced by COVID-19 at the top. The report highlights the progress made and challenges that lie ahead for the ambitious Sustainable Development Goals for 2030. Globally, the net reduction in the TB incidence rate between 2015 and 2023 was 8.3%, far from the WHO End TB Strategy milestone of a 50% reduction by 2025. The WHO South-East Asia Region accounts for more than 45% of the burden of annual incidence of TB, with six countries contributing the largest number of patients with TB and TB-related deaths globally. According to the WHO report, India achieved an 18% reduction in TB incidence between 2015 and 2023, far short of achieving its own goal of eliminating TB by 2025. India still carries the highest burden of TB in the world.

With more than 10 million people infected by TB globally every year, it is estimated that about a quarter of the global population has been infected with TB. In 2023 alone, 8.2 million people were reported as having newly diagnosed infections (an increase of nearly 10% from 7.5 million in 2022). There is also a huge gap between reported and estimated incidence, with almost half of this gap accounted for by five countries in Asia: India (16%), Indonesia (11%), Pakistan (7.8%), China (6.5%), and Myanmar (6.5%). This gap remains a key weak point in the management cascade for TB, and is mainly attributed to low penetration of, and access to, diagnostic services at the point of care, despite significant investments in laboratory-systems strengthening in recent years, along with efforts at community engagement towards health-seeking behaviour.

Looking beyond the provisioning of early and accurate diagnosis with appropriate medication, and nutritional supplementation during the treatment phase, the people who are disproportionately affected by TB live in poor conditions, and require systemic interventions such as nutrition security, improved education, gainful employment, and access to quality assured primary health care. People are also pushed to bear the consequences of accessing privatised health-care channels, which manifests early and devastatingly in the form of out-of-pocket expenses. This is another example of inequity that needs to be addressed.

The *State of Inequality: HIV, Tuberculosis and Malaria* report underlines the worsening inequalities across most aspects of the disease. The odds of developing TB were nearly five times greater in urban slum environments compared with national situations overall. This report also underlines the need for complete, timely, and reliable data to overcome the limitations in our understanding of these inequalities. Such understanding is important to target interventions to the most underserved groups using population-based surveys, national TB inventory studies, and up-to-date cause-of-death data from national or sample registration systems of high quality and coverage. Indonesia is an exemplar in this regard, having set up a national TB inventory and leveraged data for its advances.

Compared with the progress made in HIV and Malaria control and elimination efforts, the trajectory towards TB elimination remains fraught, and with current strategies, few countries in the South-East Asia Region might still be able to reach SDG targets. Countries such as Sri Lanka have demonstrated success in eliminating malaria despite political and economic crisis, by utilising its primary health-care system and involving civil society. The remarkable success in achieving some of the HIV goals was driven by civil society, with non-governmental organisations reaching out to historically marginalised and often criminalised and imprisoned communities such as sex workers, men who have sex with men and transgenders, and people who inject drugs.

However, with TB, targeted interventions for marginalised populations are often less useful because of a lack of reliable guiding data for designing and deploying them and subsequently evaluating their effectiveness. In addition, early and universal use of near point-of-care molecular diagnostics is constrained by budgetary considerations, and civil society involvement is limited due to the same lack of funding. While the Global Fund has allocated resources for interventions targeted at key populations for malaria and HIV, however, it does not have an equivalent resource allocation for TB. If the data and resource gaps for rolling out and scaling up TB-targeted interventions are addressed through collaborative measures, TB control and elimination targets can still be achieved in many countries in the region, or in many parts of a large country like India, within the SDG timeframe.

Despite recent moves to scale up newer diagnostics, cheaper and shorter therapeutic regimens, newer vaccines, and nutritional supplementation, the question remains whether they are sufficient to help reach our goals to eliminate TB by 2030. The core of our future approach to eliminate TB should rely more on addressing the gaps in service delivery (point-of-care tests, tests in sufficient numbers, tests of newer type that detect breakdown of latent infection or recent infection), outreach-based diagnosis, accelerated vaccine research and development, involvement of civil society and not-for-profit organisations in increasing diagnoses, and helping people in navigating the health services delivery system, particularly targeting groups that are most marginalised and stigmatised. We need sharper strategies that ensure justice, equity, and accessibility. It would be unfortunate to not apply equity-based targeted interventions and increase resource allocation for TB—approaches that have helped significantly reduce malaria and HIV in the region. The fight against TB is as much about addressing inequities as it is about delivering care. Without bridging these gaps, progress will remain elusive.

